# The CD200-CD200 Receptor Inhibitory Axis Controls Arteriogenesis and Local T Lymphocyte Influx

**DOI:** 10.1371/journal.pone.0098820

**Published:** 2014-06-04

**Authors:** Pleunie van den Borne, Tomasz P. Rygiel, Ayla Hoogendoorn, Geertje H. A. Westerlaken, Louis Boon, Paul H. A. Quax, Gerard Pasterkamp, Imo E. Hoefer, Linde Meyaard

**Affiliations:** 1 Laboratory of Experimental Cardiology, University Medical Center, Utrecht, The Netherlands; 2 Laboratory for Translational Immunology, Department of Immunology, University Medical Center Utrecht, Utrecht, The Netherlands; 3 Department of Immunology, Center of Biostructure Research, Medical University of Warsaw, Warsaw, Poland; 4 Bioceros BV, Utrecht, The Netherlands; 5 Department of Surgery, Leiden University Medical Center, Leiden, The Netherlands; 6 Einthoven Laboratory of Experimental Vascular Medicine, Leiden University Medical Center, Leiden, The Netherlands; 7 Interuniversity Cardiology Institute of the Netherlands, Utrecht, The Netherlands; Cardiological Center Monzino, Italy

## Abstract

The role of the CD200 ligand-CD200 receptor (CD200-CD200R) inhibitory axis is highly important in controlling myeloid cell function. Since the activation of myeloid cells is crucial in arteriogenesis, we hypothesized that disruption of the CD200-CD200R axis promotes arteriogenesis in a murine hindlimb ischemia model. Female *Cd200^−/−^* and wildtype (C57Bl/6J) mice underwent unilateral femoral artery ligation. Perfusion recovery was monitored over 7 days using Laser-Doppler analysis and was increased in *Cd200^−/−^* mice at day 3 and 7 after femoral artery ligation, compared to wildtype. Histology was performed on hindlimb muscles at baseline, day 3 and 7 to assess vessel geometry and number and inflammatory cell influx. Vessel geometry in non-ischemic muscles was larger, and vessel numbers in ischemic muscles were increased in *Cd200^−/−^* mice compared to wildtype. Furthermore, T lymphocyte influx was increased in *Cd200^−/−^* compared to wildtype. CD200R agonist treatment was performed in male C57Bl/6J mice to validate the role of the CD200-CD200R axis in arteriogenesis. CD200R agonist treatment after unilateral femoral artery ligation resulted in a significant decrease in vessel geometry, perfusion recovery and T lymphocyte influx at day 7 compared to isotype treatment. In this study, we show a causal role for the CD200-CD200R inhibitory axis in arteriogenesis in a murine hindlimb ischemia model. Lack of CD200R signaling is accompanied by increased T lymphocyte recruitment to the collateral vasculature and results in enlargement of preexisting collateral arteries.

## Introduction

Cardiovascular disease and its resulting morbidity and mortality are still a major health problem in the modern Western world. It is often associated with vascular occlusion resulting in local tissue ischemia. Stimulating perfusion recovery after vascular occlusion may be beneficial for many patients suffering from peripheral artery disease. As a response to local tissue ischemia, the human body is capable to restore blood flow with the adaptive growth of pre-existing collateral arteries into larger conduit arteries. This process is known as arteriogenesis [1], [2] Circulating inflammatory cells can extravasate from the bloodstream into the tissue and recruitment and proliferation of vascular smooth muscle cells have been shown to be of importance during arteriogenesis [3]. Migration of vascular smooth muscle cells and outward growth of the collateral vessel is enabled by disruption of the extracellular matrix by matrix metalloproteiases. Recruited inflammatory cells start to produce cell-attracting substances (chemokines). These chemokines have shown to play a crucial role in the process of local recruitment of inflammatory cells as monocytes, macrophages for stimulation of arteriogenesis, but has also been indicated to affect migration and proliferation of VSMCs locally. In addition to monocytes [1], [4]–[7], T lymphocytes (cytotoxic T cells, T helper cells and Natural Killer T cells) have been shown to contribute to arteriogenesis [8]–[10].

The main role of the immune system is to protect against different pathogens by an adequate immune response. However, damage may result from inappropriate activation of the immune system. The CD200-CD200 receptor (CD200R) axis is known as an inhibitory axis, critical in controlling excessive inflammatory responses in the case of infection or inflammation [11], [12]. CD200 is a membrane glycoprotein expressed by a wide range of cells, including neurons, endothelium, smooth muscle cells and immune cells, such as T lymphocytes, B lymphocytes and dendritic cells [13]–[16]. In contrast, expression of CD200R is restricted to lymphoid cells, such as T lymphocytes, B lymphocytes, Natural Killer cells and myeloid cells, including dendritic cells, mast cells, eosinophils, basophils, neutrophils and macrophages, particularly the M2a subpopulation [15], [17], [18]. Ligation of CD200R by CD200 has immunomodulatory effects, such as induction of immune tolerance, regulation of cell differentiation, adhesion and chemotaxis of various cell populations [19]. Furthermore, CD200R ligation is involved in cytokine and chemokine release from leukocyte subsets [11]. Mice lacking CD200 (*Cd200^−/−^*) revealed the importance of CD200-CD200R interactions in dampening the immune system. *Cd200^−/−^* mice have an increased sensitivity to autoimmune diseases, such as encephalomyelitis and collagen induced arthritis, compared to wildtype controls [12]. We previously showed that mice lacking CD200 suffer from increased immunopathology in response to influenza virus infections, compared to wildtype controls [20], for which T lymphocytes are essential. On the other hand, the absence of CD200-CD200R signaling breaks tumor tolerance and inhibits outgrowth of endogenous tumors [21].

We hypothesized that the CD200-CD200R axis is involved in arteriogenesis. Disruption of the axis might lead to more activation of the immune system during hindlimb ischemia resulting in stimulation of arteriogenesis. In this study, we investigated perfusion recovery in *Cd200^−/−^* mice as well as after CD200R ligation *in vivo* in a murine hindlimb ischemia model and assessed histological features of arteriogenesis. We observed that mice lacking CD200 have larger collateral vessels and increased perfusion recovery after hindlimb ischemia. This was accompanied by significant increase in local T lymphocyte influx compared to wildtype. In addition, ligating CD200R *in vivo* by CD200R agonist treatment in wildtype mice resulted in significantly smaller collateral vessels and a lower perfusion recovery after hindlimb ischemia. This was accompanied by a decreased T lymphocyte influx compared to isotype treatment.

## Methods

### Animal procedures

The present study was approved by the Utrecht University animal experimental committee following the Guide for the Care and Use of Laboratory Animals published by the US National Institute of Health (NIH Publication No. 85–23, revised 1996).

For the *Cd200^−/−^* study, female C57Bl/6J (wildtype) and *Cd200^−/−^* (C57Bl/6J background) mice were bred at the Utrecht University until age 10–14 weeks before undergoing unilateral permanent femoral artery ligation as described earlier [7]. For the CD200R agonist study, male wildtype C57Bl/6J mice were ordered from Charles River (age 10–12 weeks) and also underwent unilateral permanent femoral artery ligation as described earlier [7], followed by a two times intravenous injection of OX110 (a kind gift of Neil Barclay, Oxford) or rat IgG_2a_ control (anti-βGal, GL117) both at a dose of 100 µg/injection, immediately after surgery and at day 2.

Prior to surgery, all mice were anaesthetized with midazolam (2 mg/kg) and medetomidine (0.15 mg/kg) and analgesized with fentanyl (0.02 mg/kg) through a single intraperitoneal injection. After surgery, the anaesthetics were antagonized by a single subcutaneous injection of flumazenil (0.8 mg/kg) and atipamezole (4.0 mg/kg). After operation the mice received two injections of buprenorphine (0.15 mg/kg) via subcutaneous injection. Paw perfusion was assessed using Laser-Doppler Perfusion Imaging (LDPI) (Moor Instruments, Ltd, Devon, UK) before and after surgery. In addition, paw perfusion was assessed at day 3 and 7, without the use of fentanyl to avoid potential changes in vasotonus. Perfusion recovery in the ischemic limb is expressed as a percentage of the contralateral non-ischemic limb perfusion. Muscle tissue was isolated for immunohistochemistry. For the *Cd200^−/−^* study, mice were terminated at baseline, day 3 and day 7. After CD200R agonist treatment, all mice were terminated at day 7. Termination was performed by an overdose of euthesate mix with ketamine and midazolam (5 mg/kg; 0.15 mg/kg) via intraperitoneal injection.

### Immunohistochemistry

Immunohistochemistry was performed on the adductor and peroneus muscle at baseline, day 3 and/or day 7 after surgery. Muscles were embedded in paraffin and 4 µm thick sections were cut. Prior to staining, all sections were boiled in Sodium Citrate Tribasic Hydrate (10 mM, pH 6.0) as antigen retrieval solution. Tissue T lymphocytes were identified by staining for CD3 (DAKO, A0452), followed by Powervision α-rabbit-HRPO (ImmunoVision Technologies, DPVM-110HRP). Tissue macrophages were identified by staining for Mac-3 (BD Pharmingen, 550292), followed by biotin labeled goat α-rat (Southern Biotech, 3050-08) and streptavidin-HRPO (Southern Biotech, 7100-05). All sections were developed using 3-Amino-9-ethylcarbazole (AEC) and counterstained using hematoxylin. To assess alpha-Smooth Muscle Actin (α-SMA) positive vessels in adductor tissue, the sections were incubated with a FITC-labeled α-SMA antibody (Sigma Aldrich, F3777). Nuclei were counterstained with Hoechst. In the peroneus tissue, vessels were identified by incubation with CD31 (Santa Cruz Biotechnologies, sc-1506), followed by a biotin labeled goat α-rabbit antibody (Vector, BA-1000) and streptavidin-Alexa 555 (Invitrogen, S-21381). Total number α-SMA positive vessels per section were counted and CD31 positive vessels were counted per frame and corrected for muscle fiber number (CD31 positive vessels per muscle fiber). T lymphocyte and macrophage numbers were counted in the perivascular space of three to six randomly chosen collateral vessels per section and calculated as average per vessel. All immunohistochemical stainings were analyzed in a blinded fashion using Cell∧P analysis software (Olympus).

### Collateral vessel size measurements

Vessel wall dimensions and lumen area of collateral vessels were assessed at baseline, day 3 and/or day 7 after surgery from three to six randomly chosen α-SMA positive cross-sectional vessels per section and averaged per vessel. Maximal vasodilation was assured by flushing with a vasodilator (nitro-glycerine) during perfusion fixation of the hindlimbs. Measurements were analyzed using Cell∧P analysis software (Olympus). Vessel wall area and lumen area are expressed in square micrometer (µm^2^); vessel wall thickness, outer and inner perimeter in micrometer (µm).

### Flow Cytometry

Whole blood analysis was performed using flow cytometry to analyze leukocyte composition 3 and 7 days after surgery in wildtype and *Cd200^−/−^* mice. Heparinized whole blood was stained with fluorescent-labeled antibodies. The following antibodies were used: CD3-PE (T lymphocytes, eBiosciences), Ly6C-PacificBlue (monocytes, eBiosciences) and CD11b-PE/Cy7 (eBiosciences) as a marker for the activation status. Data was analyzed using Kaluza software (Beckman Coulter Inc.) and T lymphocyte and monocyte numbers are expressed in percentage of total leukocytes. Ly6Chigh and Ly6Clow monocyte numbers are expressed in percentage of total monocytes. CD11b expression is expressed in mean fluorescent intensity.

### Statistical analysis

IBM SPSS statistics version 20 (New York, USA) was used for statistical analysis. Statistical differences between two groups (*Cd200^−/−^* versus wildtype or CD200R agonist versus IgG) were analyzed using Mann-Whitney U test. Differences with P-values <0.05 were considered significant. Data were expressed as mean ± Standard Error of the Mean (SEM).

## Results

### Collateral vessel geometry and perfusion recovery after arterial occlusion

**Figure 1:**
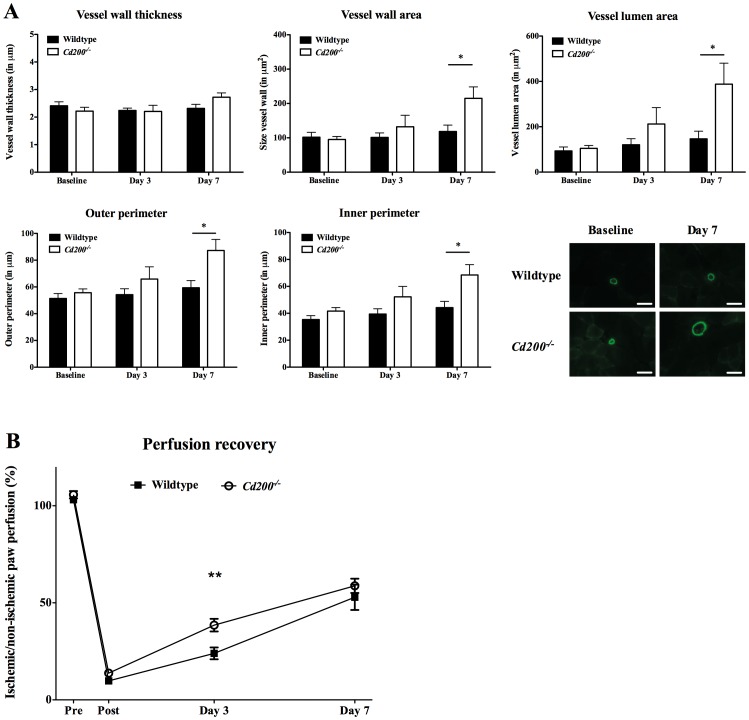
Collateral vessel geometry and perfusion recovery after arterial occlusion in wildtype and *Cd200^−/−^* mice. Collateral vessel geometry was performed at baseline, day 3 and day 7 in wildtype (black bars) and *Cd200^−/−^* mice (white bars) (A). Vessel wall thickness is calculated in the average diameter of the vessel wall and expressed in micrometer (µm). Vessel wall area per vessel is expressed in square micrometer (µm^2^) and calculated as area of total vessel minus lumen area. Lumen area per vessel is expressed in square micrometer (µm^2^). Outer and inner perimeter is expressed in micrometer (µm). Photos are representative images of alpha-Smooth Muscle Actin (**α**-SMA) staining for wildtype and *Cd200^−/−^* mice at baseline and day 7. Scale bar indicates 100 µm. Number of animals: n = 9–11 per time point for both wildtype mice (black bars) and *Cd200^−/−^* mice (white bars). Perfusion recovery was performed at baseline, day 3 and day 7 and was calculated as percentage perfusion in operated (ischemic) of the control (nonischemic) paw (B). Number of animals: n = 35 at baseline, n = 25 at day 3, n = 23 at day 7 for wildtype mice (black squares) and n = 35 at baseline day 3, n = 26 at day 7 for Cd200−/− mice (white circles). Data are presented as mean ± Standard Error of the Mean (SEM); *P<0.05; **P = 0.001 (*Cd200^−/−^* versus wildtype).

A hallmark of arteriogenesis is the enlargement of preexisting collaterals that bypass the occluded naïve artery in response to tissue ischemia [1], [22], [23]. Following unilateral hindlimb ischemia, collateral vessel geometry was measured in adductor tissue from wildtype and *Cd200^−/−^* mice. Parameters defining collateral vessel geometry were similar in both groups at baseline, but increased over time in wildtype and *Cd200^−/−^* mice. This increase was larger in *Cd200^−/−^* mice and most prominent at day 7 (P = 0.067 for vessel wall thickness, P = 0.020 for vessel wall area, P = 0.010 for vessel lumen area, P = 0.016 for outer perimeter and P = 0.010 for inner perimeter) (figure 1A). Perfusion recovery was measured using Laser-Doppler analysis. *Cd200^−/−^* mice show a significantly higher perfusion recovery compared to wildtype at day 3 after surgery (*Cd200^−/−^* versus wildtype: 39%±3.3 versus 24%±3.0; P = 0.001). This difference remained visible throughout day 7, although not significantly different (*Cd200^−/−^* versus wildtype: 59%±3.7 versus 53%±6.6; P = 0.100) (figure 1B).

### Vessel number in adductor and peroneus tissue

**Figure 2:**
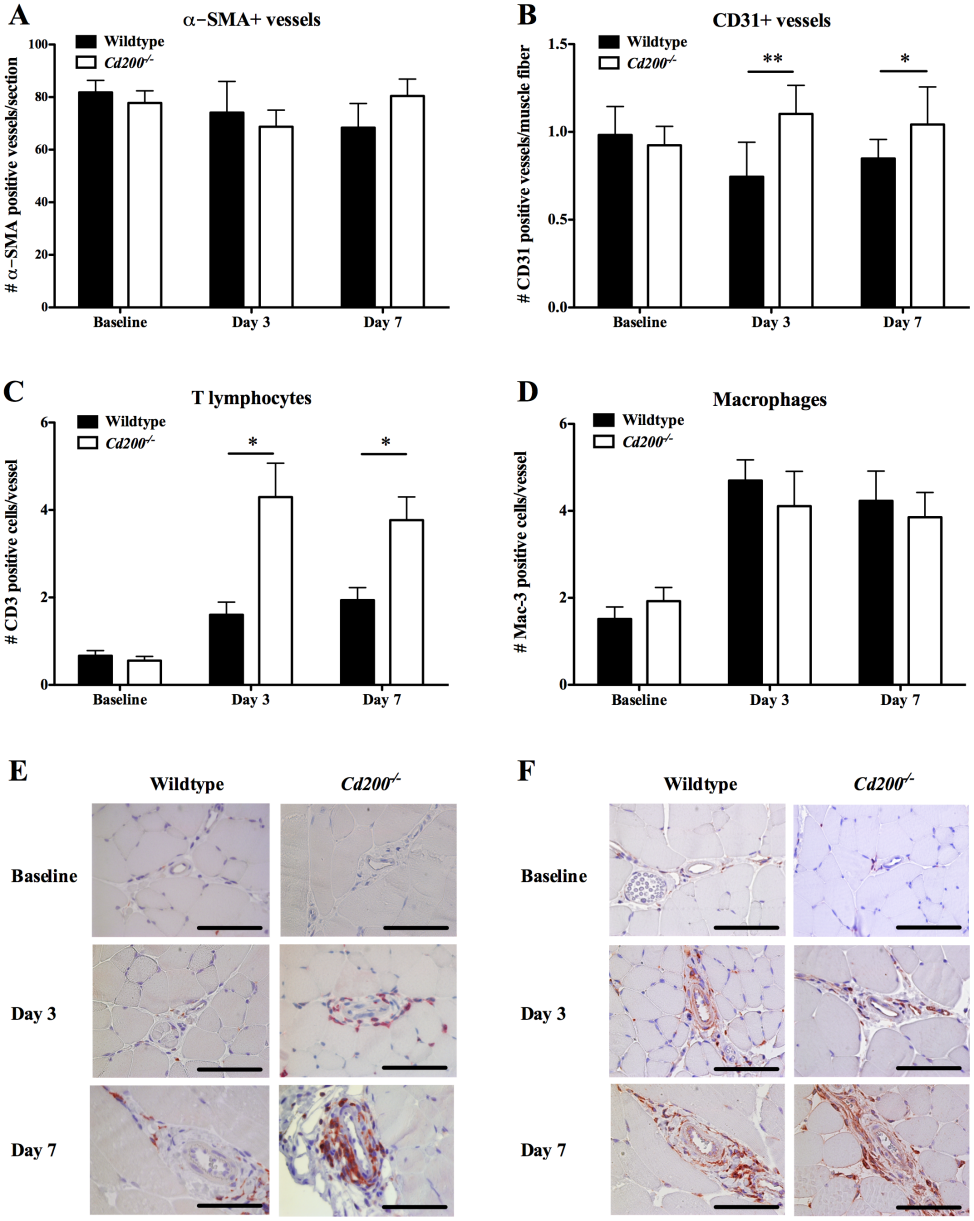
Histology for vessel numbers and inflammatory cell influx in wildtype and *Cd200^−/−^* mice. Histology was performed at baseline, day 3 and day 7 in wildtype (black bars) and *Cd200^−/−^* mice (white bars). Total number of alpha-Smooth Muscle Actin (**α**-SMA) positive vessels per section in the adductor muscle of the operated hindlimb (n = 9–11 per time point and genotype) (A). Number of CD31 positive vessels corrected for muscle fiber number in the ischemic peroneus muscle of the operated hindlimb (n = 9–11 per time point and genotype) (B). T lymphocyte (C) and macrophage number (D) was determined in the perivascular space around growing collateral vessels in the adductor muscle of the operated hindlimb (n = 9–11 at baseline and day 3; n = 23–26 at day 7, both wildtype and *Cd200^−/−^*). Photos are representative images of CD3 (E) and Mac-3 (F) staining. Scale bar indicates 100 µm. Data are presented as mean ± Standard Error of the Mean (SEM). *P = 0.01 for T lymphocytes; *P = 0.02, **P<0.001 for CD31 positive vessels (*Cd200^−/−^* versus wildtype).

During perfusion recovery, not only collateral vessel enlargement occurs, but also the numbers of arterioles and capillaries can increase. We assessed the number of α-SMA positive vessels in the adductor muscle (thigh muscle) of the operated hindlimb (total number of α-SMA positive vessels per section), defining arteriogenesis. This number did not increase in time and was not different between *Cd200^−/−^* and wildtype mice (figure 2A).

Although arteriogenesis in the used mouse model occurs in the non-ischemic upper hindlimb muscle (adductor), angiogenesis is restricted to the ischemic lower hindlimb muscle (peroneus), that is, distal from the occlusion. In the peroneus muscle, we assessed the number of CD31 positive vessels, expressed per muscle fiber in the operated hindlimb. At baseline, no differences between wildtype and *Cd200^−/−^* were observed. After surgery, an increased number of CD31 positive vessels per muscle fiber was observed in *Cd200^−/−^*, but not in wildtype mice, resulting in a marked difference as soon as day 3 (P<0.001 at day 3; P = 0.020 at day 7) (figure 2B).

### Influx of inflammatory cells

Both influx of T lymphocytes and macrophages is important during vascular remodeling and perfusion recovery [3], [9]. Therefore, we quantified both cell types in the perivascular space of collateral conduit vessels in the adductor muscle tissue at different time points after surgery. T lymphocyte influx in the perivascular space was larger in *Cd200^−/−^* mice compared to wildtype mice, resulting in a significant difference at day 3 (*Cd200^−/−^ versus* wildtype: 4.3±0.8 *versus* 1.6±0.3, P = 0.010) and 7 (*Cd200^−/−^ versus* wildtype: 3.8±0.5 *versus* 1.9±0.3, P = 0.012) (figure 2C). Macrophage numbers also increased as soon as day 3, but did not differ between wildtype and *Cd200^−/−^* mice and remained at similar levels at day 7 in both groups (figure 2D).

### Leukocyte composition in blood

**Figure 3:**
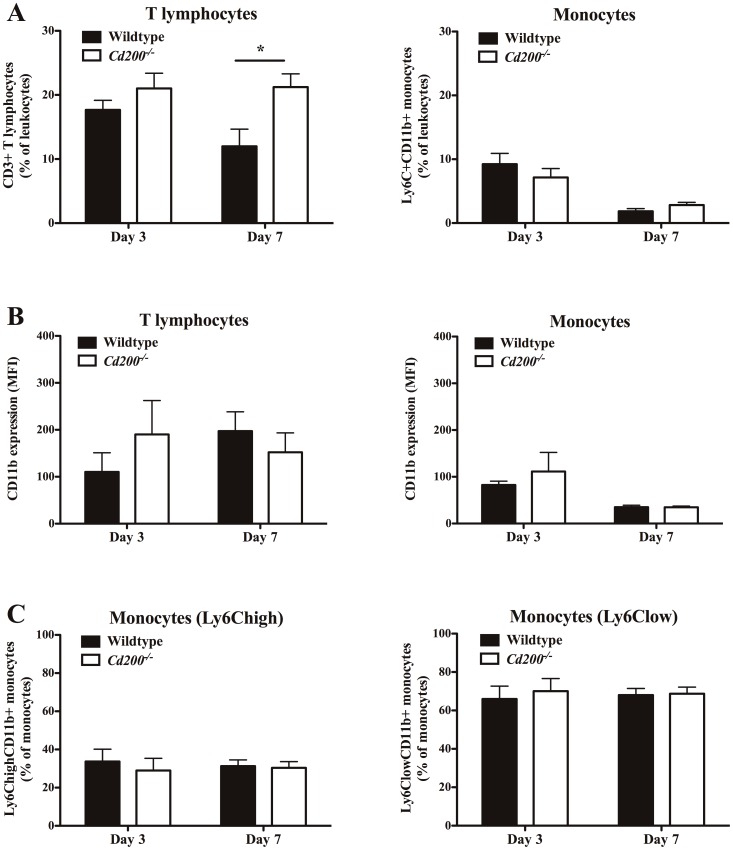
Whole blood leukocyte composition after femoral artery ligation in wildtype and *Cd200^−/−^* mice. Leukocyte composition of whole blood was analyzed at day 3 and day 7 in wildtype (black bars) and *Cd200^−/−^* mice (white bars) using flow cytometry (n = 9–11 per time point and genotype). CD3+ T lymphocytes and Ly6C+CD11b+ monocytes are expressed in percentage of total leukocytes after erythrocyte lysis (A). Activation status of CD3+ T lymphocytes and Ly6C+CD11b+ monocytes was analyzed using the CD11b expression (mean fluorescent intensity) (B). Changes in monocyte subsets were analyzed using expression levels of Ly6C (high or low mean fluorescent intensity) on Ly6C+CD11b+ monocytes. Numbers of Ly6ChighCD11b+ monocytes and Ly6ClowCD11b+ monocytes are expressed as a percentage of total monocytes. Data are expressed as mean ± Standard Error of the Mean (SEM); * P = 0.02 (wildtype vs *Cd200^−/−^*).

At day 3 and 7 after surgery, leukocyte composition of whole blood was analyzed using flow cytometry. At day 3, no differences could be observed in T lymphocyte percentages between wildtype and *Cd200^−/−^* mice. At day 7, T lymphocyte percentage in *Cd200^−/−^* remains at a similar level, whereas in wildtype mice this percentage drops (21%±2.1 vs 12%±2.7; P = 0.02). Monocyte numbers drop at day 7 compared to day 3, in both wildtype and *Cd200^−/−^* mice and levels do not differ between the two genotypes (figure 3A). CD11b expression, a marker for cell activation, in both T lymphocytes and monocytes, does not show dramatic differences in time and between wildtype and *Cd200^−/−^* mice (figure 3B). In addition, no differences were observed for the monocyte subsets (Ly6Chigh and Ly6C low) as expressed as percentage of total monocyte count (figure 3C).

### Collateral vessel geometry and perfusion recovery after CD200R stimulation

**Figure 4:**
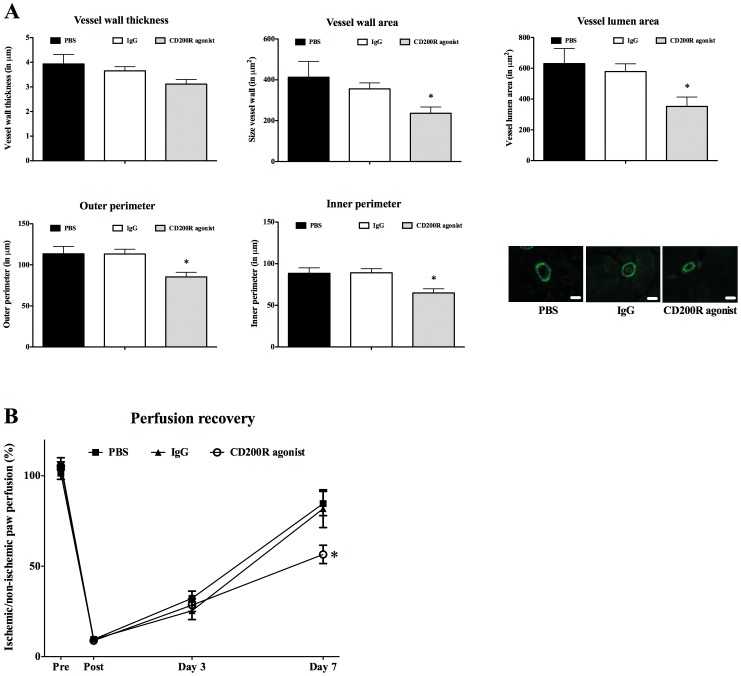
Collateral vessel geometry and perfusion recovery after arterial occlusion followed by CD200R agonist treatment in wildtype mice. Collateral vessel geometry was performed after PBS (black bars) IgG (white bars) and CD200R agonist (grey bars) treatment (n = 10 per treatment) (A). Vessel wall thickness is calculated in the average diameter of the vessel wall and expressed in micrometer (µm). Vessel wall area per vessel is expressed in square micrometer (µm^2^) and calculated as area of total vessel minus lumen area. Lumen area per vessel is expressed in square micrometer (µm^2^). Outer and inner perimeter is expressed in micrometer (µm). Photos are representative images of alpha-Smooth Muscle Actin (α-SMA) staining for PBS, IgG and CD200R agonist treatment at day 7. Scale bar indicates 100 µm. Number of animals: n = 10 for PBS (black bar), IgG (white bar) and CD200R agonist (grey bar). Perfusion recovery was performed at baseline, day 3 and day 7 and was calculated as percentage perfusion in operated (ischemic) of the control (nonischemic) paw (B). Number of animals: n = 10 for PBS (black square), IgG (black triangle) and CD200R agonist (white circle) at all time points. Data are presented as mean ± Standard Error of the Mean (SEM); * P<0.05 (CD200R agonist versus IgG).

**Figure 5:**
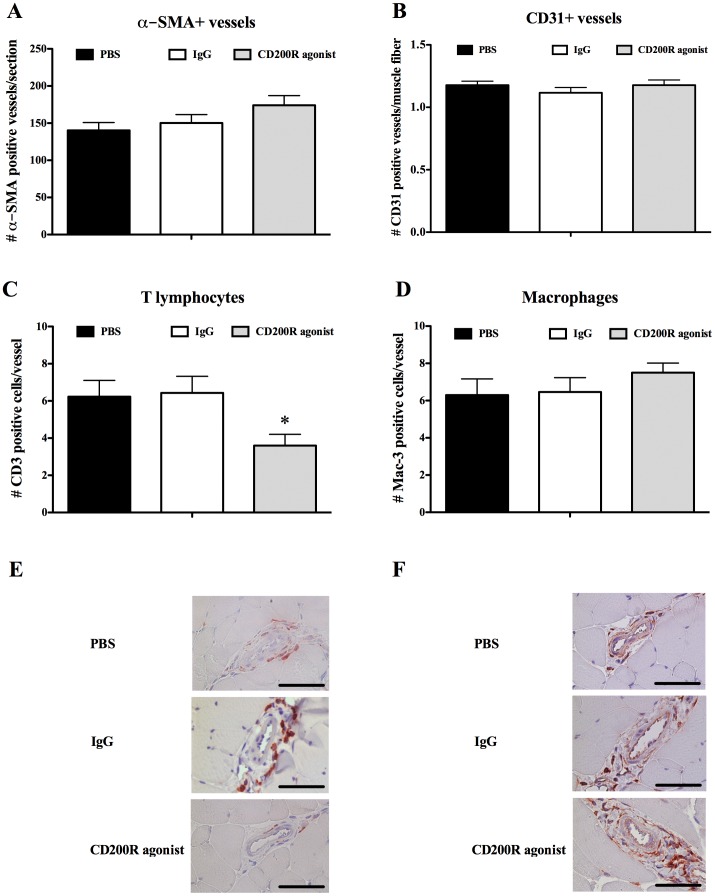
Histology for vessel numbers and inflammatory cell influx after CD200R agonist treatment in wildtype mice. Histology was performed after PBS (black bars), IgG (white bars) or CD200R agonist (grey bars) treatment (n = 10 per treatment) at day 7. Total number of alpha-Smooth Muscle Actin (**α**-SMA) positive vessels per section in the adductor muscle of the operated hindlimb (A). Number of CD31 positive vessels corrected for muscle fiber number in the ischemic calf muscle of the operated hindlimb (B). T lymphocyte (C) and macrophage number (D) was determined in the perivascular space around growing collateral vessels in the adductor muscle of the operated hindlimb. Photos are representative images of CD3 (E) and Mac-3 (F) staining. Scale bar indicates 100 µm. Data are presented as mean ± Standard Error of the Mean (SEM). *P = 0.03 (CD200R agonist versus IgG).

To validate the role of the CD200-CD200R axis in arteriogenesis, we investigated if triggering the CD200R adversely affects arteriogenesis after hindlimb ischemia in wildtype mice. Following unilateral hindlimb ischemia, C57Bl/6J mice were treated (day 0 and 2 via intravenous injection) with a specific CD200R agonist (OX110). Treatment with a rat IgG_2a_ control antibody (anti-βGal) served as an isotype control. Perfusion recovery was monitored using Laser-Doppler analysis. Histological analysis was performed on the hindlimb muscles at day 7. Collateral vessel geometry at day 7 was lower after CD200R agonist treatment compared to IgG treatment (P = 0.035 for vessel wall thickness, P = 0.015 for vessel wall area, P = 0.011 for vessel lumen area, P = 0.007 for outer perimeter and P = 0.003 for inner perimeter) (figure 4A). In line with this, perfusion recovery was also significantly lower at day 7 after CD200R agonist treatment (CD200R agonist versus IgG; 56%±5.0 versus 84%±9.7, P = 0.026) (figure 4B). Number of α-SMA and CD31 positive vessels was unaffected at day 7 after CD200R agonist treatment compared to IgG treatment (***figure 5A and B*** **)**.

### Influx of inflammatory cells after CD200R stimulation

Histological analysis at day 7 revealed a decrease in T lymphocyte influx after CD200R agonist treatment compared to IgG treatment (CD200R agonist versus IgG; 6.4±0.9 versus 3.6±0.6, P = 0.029) (figure 5C), without affecting local macrophage influx (figure 5D). This is in line with the data obtained in the *Cd200^−/−^* mice and underlines the importance of the CD200-CD200R axis in the process of arteriogenesis.

## Discussion

The process of collateral artery formation (arteriogenesis) after local arterial occlusion is known as a tightly regulated inflammatory process, in which local influx of inflammatory cells is a necessity. The CD200-CD200R axis has been identified as an inhibitory axis to restrain inflammatory responses and its exacerbation after infection. Its involvement in arteriogenesis has yet to be elucidated.

To investigate the role of CD200-CD200R axis in arteriogenesis, *Cd200^−/−^* mice subjected to unilateral femoral artery ligation, followed by analysis of collateral vessel geometry, a hallmark of arteriogenesis [1], [22], [23]. We observed greater vessel enlargement accompanied by an increased perfusion recovery, compared to wildtype mice. These results were validated by CD200R agonist treatment, resulting in opposing effects regarding vessel geometry and perfusion recovery. Taken together, these findings prove causality between CD200-CD200R axis and arteriogenesis.

Unchallenged *Cd200^−/−^* mice are healthy. However, challenging these mice, for instance by viral and bacterial infections, induced tumor formation and induced autoimmunity, leads to overt phenotypes. [20], [21], [24] CD200-CD200R axis also affects Toll-like receptor 2 and 4 responses, and thereby facilitates protection against excessive inflammatory responses [25], [26]. In the past, we have shown that Toll-like receptor 2 and 4 play a crucial role in arteriogenesis [27].

Female wildtype mice have a decreased perfusion recovery compared to male wildtype mice with the similar genetic background [28]. Therefore, the window of improvement of perfusion recovery is larger in female mice, while a decreasing perfusion recovery is easier to observe in male mice, explaining our choice for female and male mice in our different experimental settings.

The CD200-CD200R axis has specifically been suggested to attenuate inflammation and tissue damage during tissue ischemia [29]. Furthermore, mice deficient for CD200R show an enhanced pro-angiogenic and pro-inflammatory response in laser-induced choroidal neovascularization, which was reduced after CD200R agonist treatment. Specifically, changes in alternatively activated M2 macrophages involved in promoting angiogenesis, were observed [30]. In our study, we investigated inflammatory cell influx in the perivascular space as a response to tissue ischemia and observed a marked difference in T lymphocyte influx between *Cd200^−/−^* and wildtype mice. As expected, wildtype mice showed a temporal increase over 3 and 7 days after ischemia, whereas in *Cd200^−/−^* the T lymphocyte influx already reached its peak as early as day 3. This suggests a crucial role for CD200-CD200R axis in recruiting T lymphocytes during perfusion recovery early after the onset of tissue ischemia. In line with this, CD200R agonist treatment resulted in a marked decrease in T lymphocyte influx compared to control IgG treatment. Indeed, we found significant differences in capillary density (CD31+ vessels) in the calf. However, this difference was not due to increased numbers in *Cd200^−/−^* mice, but merely to a reduction in vessel density in the wildtype mice. In addition, after CD200R agonist treatment, no differences were observed in CD31+vessels compared to IgG treatment, suggesting a limited effect of changes on CD31+ vessel numbers on perfusion recovery.

Although CD200R is mainly advertised as a regulator of myeloid cell function, multiple studies report a prominent effect on T lymphocytes. The pathogenic outcome of influenza virus infection in *Cd200^−/−^* mice is dependent on the presence of both CD4+ and CD8+ T lymphocytes [20]. Furthermore, decreased tumor growth in *Cd200^−/−^* mice was associated with a shift in the regulatory T lymphocyte/T effector cell balance. Although, we did not measure cytokine production in *Cd200^−/−^* mice, we previously showed that *Cd200^−/−^* mice showed an increased production of Th2/Th17 specific cytokines, such as IL-6, IL-10 and IL-17 [21], which can also occur in our model. This mechanism might also explain the small effect on perfusion recovery, since both T lymphocyte subsets are mostly involved in the angiogenic response, which only shows a small contribution to perfusion recovery [31].

In addition, attraction of regulatory T lymphocytes and attenuation of T effector cell activation towards CD200 expressing skin grafts is associated with prolonged graft survival [32].

Whether the effect of CD200 on T lymphocytes is direct or indirect is not clear. In our study, local macrophage influx was not changed in *Cd200^−/−^* mice. However, by changing the quality of the myeloid compartment, T lymphocytes could indirectly be influenced by lack of CD200. Indeed, macrophages expressing CD200R were shown to inhibit T lymphocyte function by changing their cytokine production from a pro-inflammatory to an anti-inflammatory profile [33]. We analyzed the circulating leukocyte populations after hindlimb ischemia in both wildtype and *Cd200−/−* mice. These data show no difference in T lymphocyte numbers at day 3 between wildtype and *Cd200^−/−^*, but does show a decrease of circulating T lymphocytes in wildtype mice, suggesting to leave the circulation and entering the perivascular space around growing collaterals. In *Cd200^−/−^*, the T lymphocyte numbers are high at a constant level, even after entering the tissue, suggesting a saturation of tissue T lymphocytes. These constant high levels confirm the importance of T lymphocyte in the CD200-CD200R axis. Circulating monocyte subsets hardly changed in time and was not different between the genotypes. The role of CD200-CD200R axis in arteriogenesis could not be explained by differences in circulating monocyte subsets or monocyte tissue influx, since no differences were observed between wildtype and *Cd200^−/−^* mice.

CD200 is not only expressed by circulating inflammatory cells, but is also locally expressed by vascular cells, such as the endothelium and vascular smooth muscle cells [13]–[16]. Arteriogenesis is a local process in which increased shear stress activates the endothelium and local interaction of circulating inflammatory cells and vascular cells plays a crucial role [34]. Therefore, the interaction of CD200-expressing vascular cells with CD200R-expressing inflammatory cells can be of great importance in the regulation of arteriogenesis.

In conclusion, the CD200-CD200R inhibitory axis plays an important role in the process of arteriogenesis after unilateral femoral artery ligation in mice. The CD200-CD200R axis affects collateral vessel geometry during arteriogenesis. In addition, circulating T lymphocyte numbers and local T lymphocyte recruitment to the collateral vasculature were affected.
